# Long-Term Clinical Outcomes of Single Crowns or Short Fixed Partial Dentures Supported by Short (≤6 mm) Dental Implants: A Systematic Review

**DOI:** 10.1055/s-0043-1771028

**Published:** 2023-08-17

**Authors:** Sara Hashemi, Shivasadat Tabatabaei, Kimia Baghaei, Amirhossein Fathi, Ramin Atash

**Affiliations:** 1 Dental Students Research Committee, School of Dentistry, Isfahan University of Medical Sciences, Isfahan, Iran; 2 The School of Public Health, Boston University, Boston, Massachusetts, United States; 3 Dental Prosthodontics Department, Dental Materials Research Center, School of Dentistry, Isfahan University of Medical Sciences, Isfahan, Iran; 4 Department of Prosthodontics, School of Dentistry, Faculty of Medicine, Université Libre de Bruxelles, Brussels, Belgium

**Keywords:** crown, implant, review

## Abstract

Long-term clinical outcomes of short dental implants (≤6 mm) supporting single crowns or short fixed partial dentures have been reported differently in different studies and need more clarification. This systematic study evaluated the rate of bone loss (BL), the durability of implants equal to or shorter than 6 mm supporting single crowns or short fixed partial dentures, and prosthetic-related side effects during 5 years of follow-up. Five databases (PubMed, MEDLINE, Scopus, Google Scholar, and Cochrane) were electronically and manually searched for longitudinal studies with a follow-up period of 5 years or more until January 2023. The study question was, “Does the implant equal to or shorter than 6 mm affect BL and survival rate of the implant-supported prosthesis after 5 years of follow-up?”. From 752 identified articles, nine studies were selected for further evaluation. After 5 years of follow-up, most studies had more than 90% survival rate and the maximum BL was 0.54 mm. Still, in internal and external connections, these changes were not substantial. For example, screw loosening was the most common problem with implanted prostheses. Implants of 6 mm or shorter are a suitable treatment option in atrophic ridges with good durability and fewer side effects during a follow-up period of more than 5 years.

## Introduction

Dental implants are a suitable method for oral rehabilitation in edentulous people (partial or complete). 1 Using conventional implants of 10 mm length have shown acceptable results in long-term follow-up. 2 However, using 10 mm-long implants in some conditions, such as atrophy or reduced bone loss (BL), is impossible. In these cases, dentists use invasive methods such as bone reconstruction or raising the maxillary sinus floor. 3 Despite the high predictive advantage, 4 it has side effects such as the need for more surgery, more extended recovery period, and higher cost. 5 Besides new findings of using new materials for tissue regeneration, 6 7 like extracted tooth material for bone augmentation, 8 studies show that using short implants in people with bone resorption has been a reliable solution over the past few years, 9 and the high crown-implant ratio does not cause any complications. 10

According to studies, implants shorter than 10 mm are equally effective compared to standard implants with rough surfaces. Using short-coated implants in the posterior areas has side effects, BL, and the fracture rate is less. 11 12 13 In addition, short implants have debatable definitions. According to various opinions, a length less than 11, 14 10, 15 or 8 mm 5 is defined as a short implant and a length less than 6.5 mm is considered a very short implant. 16 17

Although implant-assisted restorations have a high survival and success rate in treatment, these prostheses are still subject to various complications, including biological and technical. 18 19 One of these consequences may be creating incorrect occlusal forces as an axial load. In some experimental studies 20 and the animal model, 21 the results of occlusal loading showed increased pressure on the bone surrounding the implant. However, results of long-term clinical trials have shown contradictory results, for example, some longitudinal clinical studies have suggested a combination of occlusal problems and bone resorption surrounding the implant, 22 but some studies have argued that there is no association between occlusal trauma and BL around the implant. 23 There are numerous reasons to explain these discrepancies, such as it is difficult to identify the extent and direction of occlusal pressure as a complex variable. 24 However, there is no scientific evidence of the amount of bone pressure threshold surrounding the implant, which is the endpoint of the repair and the beginning of bone resorption around the prosthesis. 25 Genetic factors can also affect bone tolerance and differ from person to person.

The crown–implant ratio (C:I) indicates the axial loading. This report uses the crown as a lever arm and conveys pressure to the bone around the implant. 26 This transmitted pressure may result in bone resorption around the implant 27 or complications in implant components.

Contrary to initial concerns, the use of ultrashort implants in clinical trials has yielded satisfactory results that, compared to conventional implants, had fewer complications in the atrophic arch during the 1-year follow-up period. 28 29

According to the conventional definition, various studies examined the use of implants shorter than 10 mm. 30 31 However, they did not report significant survival rate results compared to implants longer than 10 mm in long-term follow-ups 32 33 because implants of 6 mm or shorter than 6 mm have not been systematically evaluated over a follow-up period of more than 5 years; this study aimed to assess and identify studies that report patients' survival rates, BL rates, and prosthesis adverse events. The following hypotheses were also examined: firstly, short implants have a survival rate of 5 years, and secondly, short implants reduce the side effects of the prosthesis.

## Methods

Preferred Reporting Items for Systemic Reviews and Meta-Analyses and The assessment of multiple systematic reviews (AMSTAR) protocols were used to achieve standards for reporting systematic reviews in the search process. 34 35

## PICO

The research question was, “P” or patient, including people who have used implants of 6 mm length or below for at least 60 months. “I” or intervention was the presence of an implant with a size of 6 mm or less in the mouth. “C” or comparison was in terms of the location of the implant, the connections, and the level of the tissue and bone implants. “O” or outcome was short implant survival rate, prosthetic side effects, BL rate, tissue level and bone level comparison, and patient reports. 36

### Search Strategy and Resources of Information

Electronic databases such as MEDLINE, PubMed, Scopus, Google Scholar, and Cochrane were searched. Another manual search covering the period to 2023 was also conducted manually in dental journals. In the articles obtained from this search, the following variables were extracted: implant survival rate, the average bone surface area around the implant, and technical complications related to implant components and/or prosthetic restoration structure.

The systematic literature search was performed with the following search terms:

(1) MEDLINE: (short [All Field] AND implants [All Field] AND English [All Field] AND (Randomized Controlled Trials) AND “Humans” [Mesh Terms]); (2) Scopus: short AND implants AND Randomized Controlled Trials AND [English]; (3) Cochrane: short dental implants AND Trials

The search results were obtained in an electronic database selected by two independent researchers. Two investigators chose the studies based on the title and content of the summary. In case of disagreement, they consulted a third researcher to solve the problem. Selected papers for the evaluation were reviewed, and their reference list was concerned, after the initial review of the selected articles, due to the significant heterogeneity in the type of study design, and data collection methods between studies, at the discretion of the author, quantitative data analysis and meta-analysis were not performed. Therefore, descriptive data were analyzed.

This study only contains longitudinal prospective, retrospective, or randomized controlled trials (RCTs), which examined the length of the implant, and the average follow-up period of 5 years and was written in English.

## Result

### Study Selection

**Fig. 1:**
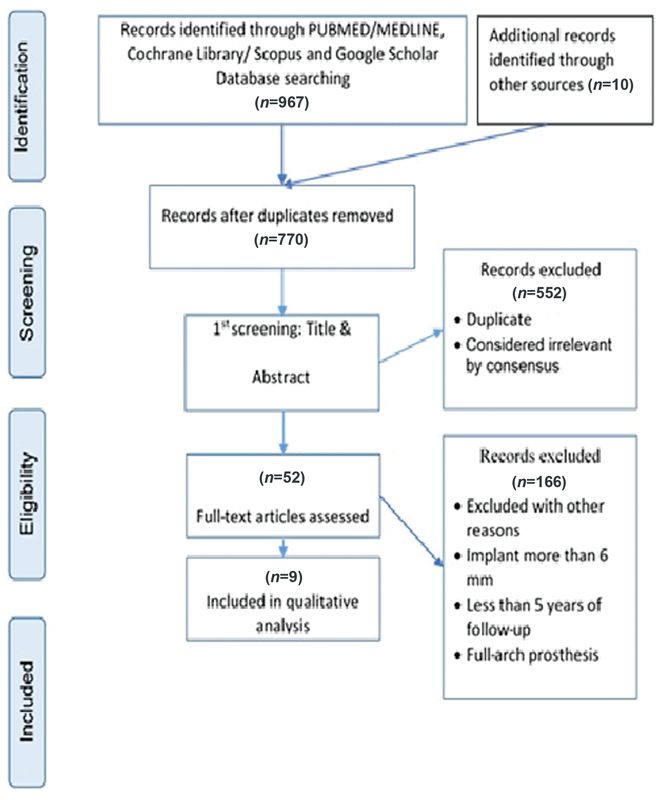
Flow charts for the studies were identified, displayed, and included in the study.

As a result of this research, 977 papers were identified, and 52 studies were chosen to examine and study the full text. Of these, only nine articles conformed to this systematic review's inclusion and exclusion criteria 37 38 39 40 41 42 43 44 45 ( Fig. 1 ).

### Quality Evaluation

**Fig. 2:**
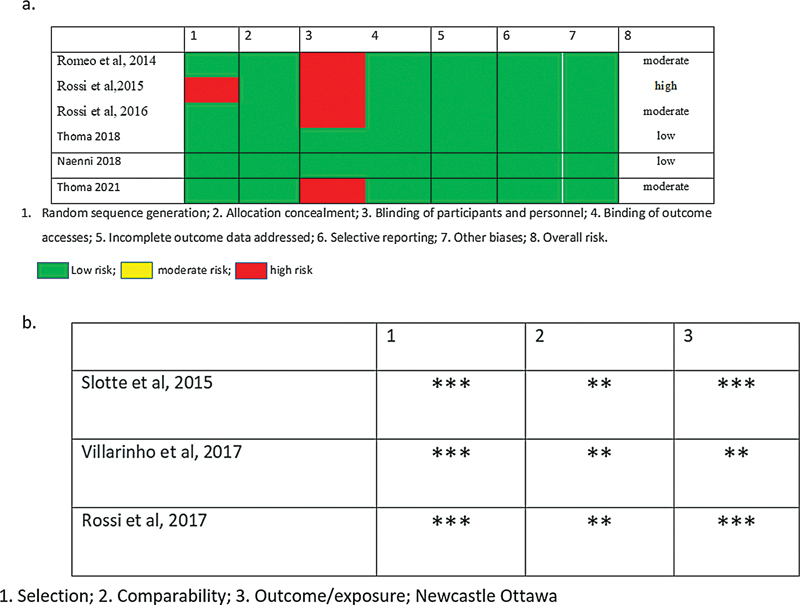
Risk of bias for ( **a** ) RCT and ( **b** ) non-RCTs.

Fig. 2 summarizes the results of the risk of bias in selected studies. Of the nine chosen studies, six were RCTs, and the three were non-RCTs. However, two RCT had a low risk of bias, three studies had a moderate risk of bias, and one study had a high risk of bias. Non-RCT studies were assessed based on the Newcastle OTAWA scale.

### Characteristics of Selected Articles

The table shows the characteristics of the selected articles. Information about the mandible and maxilla were evaluated separately. Studies had a follow-up period of 5 years ( Tables 1 and 2 ).

**Table 1:** A summary of the most important information of the selected studies

Study			Population		Implant			
	Design	Follow-up (Y)	Patients ( *n* )	Mean age (Y)	Total	Location	Length (mm)	Diameter (mm)
** Romeo et al 2014 45**	RCTs	6	24	50	26	Maxilla (5) Mandible (21)	6	Wide (26)
** Rossi et al 2015 44**	RCTs	5	35	51	40	Maxilla (15) Mandible (25) Premolars (14) Molars (26)	6	Regular (19) Wide (21)
** Slotte et al 2015 37**	Prospective	5	32	64	86	Mandible (86)	4	Regular (86)
** Rossi et al 2016 38**	RCTs	5	45	48.4	30	Maxilla (12) Mandible (18) Premolars (17) Molars (13)	6	Regular (30)
** Villarinho et al 2017 39**	Prospective cohort	6	20	52	46	Maxilla (23) Mandible (23) Premolars (12) Molars (34)	6	Regular (46)
** Rossi et al, 2017 40**	Prospective cohort	5	20	55	40	Maxilla (14) Mandible (26)	6	Regular ( *n* = 29) Wide ( *n* = 11)
** Thoma 2015 42**	RCT	5	44	50	60	Maxilla (60)	6	Regular ( *n* = 60)
** Naenni 2018 43**	RCT	5	40	58.2	40	Maxilla (12) Mandible (28)	6	Regular ( *n* = 40)
** Thoma 2021 41**	RCT	5	26	67.5	48	Maxilla (19) Mandible (29)	6	Regular ( *n* = 48)

Abbreviation: RCT, randomized controlled trial.

**Table 2:** Characteristic of included study

Study	Type of loading	Type of retention	Type of prosthesis	Type of implant	Type of connection	outcome	
			(if partial, number of units per restoration)			Failure (early: late)	Marginal bone loss in the last follow-up (mean± SD)
** Romeo et al, 2014 45**	Late	cemented	Single	Tissue level	Internal	1:0	0.4 ± 0.34 mm
** Rossi et al, 2015 44**	Late	cemented	Single	Tissue level	Internal	2:0	0.43 ± 0.49 mm
** Slotte et al, 2015 37**	Late	Screw retained	3 to 4 splinted	Tissue level	Internal	0:6	0.53 mm
** Rossi et al, 2016 38**	Late	Screw retained	Single	Tissue level	Internal	1:4	0.14 mm
** Villarinho et al, 2017 39**	Late	Screw retained	Single	Tissue level	Internal	4:0	0.3 ± 0.5 mm
** Rossi et al, 2017 40**	Early	Screw retained	2 to 3	Tissue level	Internal	0:4	0.3 ± 0.4 mm
**Thoma** et al, ** 2018 42**	Late	Screw retained	1 unit	Bone level	Internal	0:1	0.54 ± 0.87 mm
**Naenni** et al, ** 2018 43**	Late	Screw retained	1 unit	Tissue level	Internal	0:4	-0.29 mm
**Thoma** et al, ** 2021 41**	Late	Screw retained	1 unit with cantilever / 2 single unit	Bone level	internal	0:2	0.29 ± 0.63 mm 0.17 ± 0.59 mm

### Specifications of Implants in Terms of Location and Size

The number of implants shorter than 6 mm was included and examined. The total number of implants with 6 mm lengths was 416, respectively. There was not any implant with lengths below 6 mm. The typical widths were regular (358), wide (58), and 4.2 mm, respectively. Studies were performed on implant restorations with screws retained in the seven studies, 37 38 39 40 41 42 43 while cemented restorations were in the two studies. 44 45

### Bone Loss Analysis

Several studies examined the rate of BL using a standardized radiographic method, but many studies did not follow the standard form. Therefore, this systematic study evaluated the amount of BL in nine studies 37 38 39 40 41 42 43 44 45 from the implant insertion to the final follow-up.

In the first year of BL follow-up, seven studies reported BL, and two studies reported bone growth. Nevertheless, only four studies reported BL during follow-up intervals in the 5th years and three studies reported bone growth. However, due to the lack of sufficient information from the initial studies, it was impossible to analyze the number of changes in bone surface annually. For example, one study reported changes in BL at the end of the follow-up period. Therefore, a table is the only way to report bone changes during follow-up ( Table 3 ).

**Table 3:** The bone loss rate of implants during years of follow-up

**Study**	**Year 1**	**Year 2**	**Year 3**	**Year 4**	**Year 5**
**Romeo et al, 2014**	−0.18	−	−	−	−0.31
**Rossi et al, 2015**	−0.23	−0.2	−0.07	−0.1	−0.1
**Slotte et al, 2015**	−0.44	−0.13	+0.02	−	+0.02
**Rossi et al, 2016**	−0.13	−0.02	−0.03	+0.01	+0.03
**Villarinho et al, 2017**	−0.2	−0.1	−0.1	−0.2	−
**Rossi et al, 2017**	−0.2	−0.04	−0.03	−	−0.02
**Thoma** et al, **2018**	0.27	−	0.45	−	0.54
**Naenni** et al, **2018**	−0.18	−	−0.35	−	−0.29
**Thoma** et al, **2021**	0.03 0.2		0.32 0.46		0.29 0.17

Generally, during the follow-up of 5 years, the amount of BL in implants shorter than 6 mm varied between −0.54 mm (BL) and + 0.29 (bone growth). Also, most cases of BL occurred in the first year of implant placement, and in the following years, BL was decreased ( Table 3 ). While comparing implants implanted at tissue or bone level, the most BL occurred in bone level implants in the first year of follow-up. Finally, the rate of bone resorption in implants with internal and external connections was not significantly different during the1-year follow-up.

### Survival Rate

The survival rates in implants shorter than 6 mm was 96.1, 95, 93.02, 83.33, 91.30, 90, 98.33, 90, and 95.83. Most of the studies had more than 90% survival rate.

### Prosthesis Side Effects

Side effects of prostheses are shown in Table 2 . The most common complication of prostheses was screw loosening, which was reported. Subsequent complications were re-cementation and fracture in the prosthetic case.

## Discussion

Short implants are usually utilized in the posterior areas because in these areas, due to the presence of the alveolar nerve, the upper sinus, and the curvature of the tongue, using implants of average and standard length can be challenging. 46 Moreover, in the event of serious complications necessitating fixture removal, short implants would need a simpler removal method, decreasing BL and facilitating the possibility of rehabilitating the same location with a new implant. In addition, the possible use of short implants in sites able to accommodate longer fixtures may provide additional advantages in addition to their regular usage in atrophic locations. In fact, such a rehabilitative method does not include the aesthetic, practical, and physiological difficulties often associated with greater interarch distance and higher C:I. 47 In this case, using a short implant can cause fewer complications and is a good option.

However, despite positive reports on the performance of implants shorter than 6 mm, the effectiveness of these implants is still in doubt due to the lack of studies with sufficient follow-up time. These types of implants should be investigated in terms of force distribution and its effect on the surrounding bone, and their effectiveness in different types of restorations with a different number of units and different stress distribution levels should be investigated. Completeness of follow-up is a prerequisite for dependable outcome evaluation and should be disclosed systematically. 48 For this reason, the current study examines single crowns and short Fixed Partial Denture (FPDs) supported by short implant in long follow-ups to clarify the ambiguities in the long-term function of implants with a maximum length of 6 mm.

RCT studies with more than 5 years of follow-up have increased the validity and created a better image of implants shorter than 6 mm. 42 43

Long implants were used for a long time to reduce the stress on the bone around the implant and finally became a standard of care. However, this scenario is debatable due to the use of rough structures on the surface of these implants. The results of subsequent studies showed that the survival rate of implants with a length of less than 10 mm is similar to that of larger implants. A survival percentage higher than 90% is acceptable during a 5-year follow-up period and can justify its replacement. The results of a study which focus solely on upper jaw reported that the survival rate of short implants after a 5-year follow-up period was 98.33%. 42 However, in another study, the same results were obtained for implants shorter than 6 mm and conventional implants in the mandible, which is 93.02% and confirms the success of short implants in the mandible. 37 Two studies concluded that short implant supported single crowns or short FPDs showed a satisfying result. 39 40 In 2015, Rossi concluded that 6 mm implants with moderately rough surface supporting showed low marginal bone resorption while supporting single crowns. 44 The lowest survival rate was in a study by Rossi in 2016, which showed a 83.33% survival rate. In the most recent study by Thoma in 2021, short dental implants supporting 1 or 2 units showed a survival rate of 95.83%. 41

However, some researchers reported that in shorter implants, the C:I ratio is rising, and as a result, the potential for bone resorption around the implant can increase. 49 According to one study, crown length increases the risk more than implant length. The analysis carried out in this study showed that reducing the C:I index by reducing the size of the crown considerably reduces stress on the bone around the implant. 50 Moreover, the type of crown material and adhesion characteristics has to be considered. 51

More follow-up studies can be helpful in order to improve trustworthiness, as the force distribution is a crucial factor to avoid tension and deformation of oral appliances. 52 53 For example, it is better to check the presence of bruxism in cases of success. In addition, there is a need for uniform standard definitions for prosthesis side effects between studies. Finally, in future studies, it is necessary to investigate the force distribution on short implants that effectively changes bone density.

## Conclusion

Due to the limitations of this review, implants shorter than 6 mm generally showed an acceptable survival rate, which was higher in the mandible. Several studies examined bone resorption over a follow-up period of more than 5 years, and most studies examined bone resorption only in the first year. In addition, the amount of BL on the implant surface was higher than on the tissue surface, but internal and external connections did not play a role in BL. Although it was infrequent, the most common side effect of implants shorter than 6 mm was screw loosening. Also, the rate of adverse effects and prosthetic failures in nonsplinted implants was higher than in splinted.
